# The effects of adipose tissue-derived stem cells seeded onto the curcumin-loaded collagen scaffold in healing of experimentally- induced oral mucosal ulcers in rat

**DOI:** 10.22038/ijbms.2020.48698.11171

**Published:** 2020-12

**Authors:** Maryam Mardani, Azita Sadeghzadeh, Nader Tanideh, Azadeh Andisheh-Tadbir, Fatemeh Lavaee, Moein Zarei, Javad Moayedi

**Affiliations:** 1 Oral and Dental Disease Research Center, Department of Oral and Maxillofacial Medicine, School of Dentistry, Shiraz University of Medical Sciences, Shiraz, Iran; 2 Postgraduate Student, Oral and Dental Disease Research Center, Department of Oral and Maxillofacial Medicine, School of Dentistry, Shiraz University of Medical Sciences, Shiraz, Iran; 3 Stem Cells Technology Research Center, Shiraz University of Medical Sciences, Shiraz, Iran; 4 Oral and Dental Disease Research Center, Department of Oral and Maxillofacial Pathology, School of Dentistry, Shiraz University of Medical Sciences, Shiraz, Iran; 5 West Pomeranian University of Technology, Szczecin, Department of Polymer and Biomaterials Science, Al. Piastow 45, 71-311 Szczecin, Poland; 6 Diagnostic Laboratory Sciences and Technology Research Center, School of Paramedical Sciences, Shiraz University of Medical Sciences, Shiraz, Iran; 7 Center of Comparative and Experimental Medicine, Shiraz University of Medical Sciences, Shiraz, Iran

**Keywords:** ADSCs, Collagen, Curcumin, Experimental, Oral ulcers, Scaffold, Ulcer healing

## Abstract

**Objective(s):**

Various therapeutic approaches, including stem-cell-based strategies and tissue engineering, have been proposed for oral ulcerative lesions. We investigated the effects of adipose tissue-derived stem cells (ADSCs) seeded onto the curcumin-loaded collagen scaffold in the mucosal healing of oral ulcers in rats.

**Materials and Methods:**

The current experimental study was conducted on 40 male Sprague-Dawley rats. Oral ulcers were created over both sides of buccal mucosa, and the rats were randomly divided into four equal groups: 1) an untreated group (negative control); 2) Teriadent-treated group (positive control); 3) group treated with curcumin-loaded collagen scaffold; and 4) group received the ADSCs (3 × 10^6^ cells) seeded onto the curcumin-loaded collagen scaffold. Rats were sacrificed on 3rd and 7th day after ulceration for histopathological examination as well as measurement of tissue levels of myeloperoxidase (MPO), superoxide dismutase (SOD), and Interleukin-1 beta (IL-1β) activity.

**Results:**

Compared with the negative control, the tissue levels of MPO and IL-1β were significantly decreased in all treated groups (*P*<0.0001); however, the SOD activity was elevated (*P*<0.0001). The highest SOD activity as well as the lowest MPO and IL-1β levels were observed in the ADSCs-curcumin-loaded collagen scaffold group. The ulcer healing process at 3^rd^ and 7^th^ day follow-up was much more progressed in the ADSCs-curcumin-loaded collagen scaffold group in comparison with the untreated group (*P*=0.037 and *P*=0.004, respectively).

**Conclusion:**

According to the findings of this study, ADSCs seeded onto the curcumin-loaded collagen scaffold seems to have a promising potential for oral ulcer healing applications.

## Introduction

The epithelial layer of the oral mucosa protects the underlying tissues from microbial, chemical, and physical damages; however, this protective barrier can be disrupted by various factors, e.g., systemic disorders, neoplastic conditions, irradiation, and ulcers (1, 2). Oral ulcerative lesions are common findings in dental practice. They have three major forms (acute, chronic, and recurrent ulcers) and are mainly found on the lips and buccal mucosa (2-4). Oral ulcers are in direct contact with the salivary microorganisms and offer an opportunity to microflora to infect surrounding tissues and keep ulcers in an inflammatory state that can result in delayed healing and increased scar formation. Hence, the early initiation of healing may be critical for the management of oral ulcers (5, 6).

Advances in stem cell technology and tissue engineering have opened new vistas for timely and permanent closure of ulcers (7, 8). Stem cells are the progenitors and precursors of the body tissue defined as immature or undifferentiated cells (9). Adipose tissue-derived stem cells (ADSCs) are a promising type of stem cell for promoting tissue repair *via* their capacity of paracrine secretion of a broad spectrum of cytokines, chemokines, and growth factors. ADSCs are capable of unlimited or prolonged self-renewal, multipotential differentiation into various mature cell types, have constant availability, immunosuppressive properties, low immunogenicity, and cost-effectiveness. The ability of ADSCs to restore chronic ulcers has proven its superiority for therapeutic applications (10-12).

The ultimate purpose of tissue engineering is designing a suitable 3D supporting framework or scaffold, with ideal mechanical and biological properties to serve as a platform for cellular localization, adhesion, migration, differentiation, nutrient delivery, and eventually vascularization (13, 14). Scaffolds may have a natural, synthetic, or hybrid origin and are engineered *via* a multitude of approaches to easily integrate with the host tissue without the possibility of toxic or immunogenic effects (8, 15, 16). Collagen is the most abundant component of ECM and connective tissue, which is required for maintaining the structural integrity of the tissues. It can promote cell-biomaterial interactions, permitting sufficient transport of gases, nutrients, and regulatory factors to allow cell survival, proliferation, differentiation, migration, and the synthesis of proteins. Furthermore, it is involved in all stages of wound healing; hence, collagen remains the popular choice for tissue engineering approaches (17-19). However, it suffers from biological instability; therefore, a variety of methods such as chemical cross-linking or combined hybridization with natural polymers are demonstrated to reduce easy degradation of collagen and fabricate collagen-based scaffolds with superior properties (20).

For centuries, medicinal plants and their bioactive compounds have been considered as a central point of research for treating oral mucosal disorders. Curcumin is an orange-yellow active pigment obtained from a tropical herb called turmeric (*Curcuma longa* Linn). It is an effective and safe adjuvant for accelerating oral ulcer healing through several mechanisms. Topical application of curcumin in an animal model of oral ulcer enhanced the healing process, both histologically and immunohistochemically (21-23).

The potential benefits of each of the stem cells, collagen scaffold, and curcumin in the acceleration of the wound healing process have been confirmed in previous studies. Therefore, fabrication of a new scaffold containing all of these components might speed up the healing process and ultimately control stem cell activity. In this regard, the current study aimed to investigate the effects of curcumin-loaded collagen scaffolds as suitable supporting frameworks for ADSCs on mucosal healing of experimentally induced oral ulcers in a rat model.

## Materials and Methods

***Animals and Ethics***

This experimental study was performed on 40 adult male Sprague-Dawley rats (*Rattus norvegicus*) weighing 200–230 g. Prior to the experiments, all rats were allowed to acclimatize for a week at the Center of Comparative and Experimental Medicine, Shiraz University of Medical Sciences, Shiraz, Iran. Two rats were housed per cage in a standard laboratory housing condition (22±2 °C; 40–60% relative humidity; 12:12 hr light/dark cycle) with unlimited access to water and pellet diet. The protocol of this study was approved on November 03, 2019, by the local Ethics Committee of Shiraz University of Medical Sciences, Shiraz, Iran (Approval ID: IR.SUMS.DENTAL.REC.1398.129).

***Preparation of curcumin-loaded collagen scaffolds***

The commercially available type 1 collagen extracted from the rat-tail tendon was purchased from the Sivan Company (Cat# A1001, Sivan, Iran). At first, collagen was dissolved in 0.02 N acetic acid (Sigma-Aldrich, USA) and then, an increasing concentrations (5%, 10%, and 15%) of curcumin (Sigma-Aldrich, USA) were slowly added to the solution to optimize the best composition. A wide range of curcumin concentrations (5–25%) can be used as an additive material to make scaffolds (24). Ultimately, the lyophilization method was applied for 48 hr to obtain a soft porous freeze-dried collagen-based scaffold.

The mechanical properties of scaffolds were evaluated at room temperature using a tensile testing machine (Santam Co., Tehran, Iran) according to the ISO 1798 standards. Five samples in each group were prepared in the dimension of 10–50 mm (WL), placed between the clamping jaws of the machine, and pulled apart with 50 N loads by extension rate at 10 mm/min. The process was continued until the specimens fractured at a constant rate of displacement (25).

The morphology and diameters of lyophilized scaffolds were also investigated using scanning electron microscopy (Tescan Vega-3 LMU SEM). MATLAB and Image J software programs were used to measure the porosity and mean pore diameters of the scaffold *via* SEM photomicrograph (25). Fourier-transform infrared spectroscopy technique (FTIR-JASCO 6300, Japan) in the attenuated total reflectance mode spectra (400-4000 cm^-1^) with a resolution of 0.5 cm^-1^ with 32 scans was applied to evaluate the chemical structure of the curcumin-loaded collagen scaffold (26).

***Isolation, culture, and characterization of ADSCs***

Adult male Sprague-Dawley rats were deeply anesthetized by diethyl ether inhalation (Merck, Germany) *via* the respiratory route for approximately 2 min in a transparent acrylic jar and then sacrificed by cervical dislocation. After shaving the rat’s hair, sterilization of the skin, and opening the site, the abdominal and cervical adipose tissues were completely removed. Thereafter, the adipose tissue was harvested and kept in a 50 ml sterile falcon tube containing phosphate-buffered saline (PBS, pH 7.4; Gibco, Invitrogen, USA) and 1% penicillin-streptomycin (Sigma-Aldrich, USA). All culture procedures were carried out in a sterile laminar flow hood. Red blood cells were gently removed from the samples by washing with sterile PBS solution. The adipose tissue fragments were minced into small pieces (~1 mm^3^) using a scalpel blade and forceps, then immersed in a solution consisting of 0.1% collagenase type I (Gibco, Invitrogen, USA), and incubated at 37 °C in a shaking water bath for 90 min. After filtration of the digested tissue, the suspension was centrifuged for 5 min at 1,200 rpm at room temperature. The supernatant was discarded and the pellet was re-suspended in Dulbecco’s modified Eagle’s medium (DMEM, Gibco, Invitrogen, USA).

The adipose-derived cells were transferred into 25 mL culture flasks containing DMEM supplemented with 10% fetal bovine serum (FBS, Sigma-Aldrich, USA) and 1% penicillin/streptomycin. The culture flasks were then incubated at 37 °C for 48 hr in an incubator with 5% CO_2_ and a humidified atmosphere. Whenever the cells reached 70–80% confluency, non-adherent cells were discarded by washing with PBS. Adhered cells were detached by 0.25% trypsin-EDTA solution (Gibco, Invitrogen, USA) and further passaged in a fresh DMEM with the above-mentioned condition to provide large cell numbers (27). After the third passage, stem cell potency of ADSCs was examined by cellular morphology, plastic adherent properties, differentiation capacity into adipocytes and osteoblasts, and flowcytometric analysis of surface markers such as CD34, CD44, CD45, and CD90 (27). For adipogenic induction, ADSCs at 70% confluence in 6-well plates were cultivated for 3 weeks in DMEM, 15% FBS, 0.2 mM L-glutamine, 200 µM indomethacin, 100 µM L-ascorbic acid, and 100 nM dexamethasone. For osteogenic differentiation, the ADSCs were transferred into 6-well plates to reach 70% confluence. The osteogenic medium containing DMEM, 15% FBS, 100 nM dexamethasone, 10 mM glycerol 3-phosphate, and 200 µM L-ascorbic acid were added into cell-cultured plates. The culture media were changed twice a week for 3 weeks. Finally, differentiations to adipocytes and osteoblasts were assessed using Oil Red-O (Sigma-Aldrich, St. Louis, USA) and Alizarin Red (Sigma-Aldrich, St. Louis, USA) staining, respectively (28, 29).

***In vitro cell viability***

The cytotoxic effects of curcumin were investigated at the cellular level by 3-(4,5-dimethylthiazol-2-yl)-2,5-diphenyltetrazolium bromide assay (MTT; Sigma-Aldrich, USA). Briefly, ADSCs were seeded on the 96-well plates (5000 cells per well) and cultivated for 24 hr at 37 °C in a humidified atmosphere containing 5% CO_2_. After the incubation period, serial doses of curcumin were prepared and added to each column of a 96-well plate. Cells were incubated with curcumin for 24, 48, and 72 hr at the same conditions as described above. Thereafter, cells were treated with 20 µl of MTT solution (5 mg/ml), and the plate was incubated for 4 hr at 37 °C in a CO_2_ incubator. Consequently, the supernatant was removed and the MTT formazan crystals were solubilized by adding 200 µl of dimethyl sulfoxide (DMSO; Sigma-Aldrich, USA) to each well. Finally, the absorbance of the formazan product was measured at 570 nm, using a microplate reader (Floustar Omega, BMG Lab Tech, Ortenberg, Germany). All experiments were performed in triplicates and the optical density of control wells was considered as 100% viability (30).

***Experimental oral ulcers in rat***

Induction of general anesthesia was performed through an intramuscular injection of a mixture containing ketamine 10% (100 mg/kg; Alfasan, Woerden, Netherlands) and xylazine 2% (20 mg/kg; Alfasan, Woerden, Netherlands). In order to develop an experimental model of oral ulcer, circular ulcers were made over both sides of the buccal mucosa of rats by a 5-mm diameter punch. Afterward, the coating epithelium was removed by a number 15 scalpel blade (31, 32). A single operator performed all procedures to minimize the variability of ulcers. Subsequently, an ulcer surface was rinsed with 0.9% sterile saline solution (Darupakhsh, Tehran, Iran) and rats were randomly assigned into 4 groups (ten animals each). An untreated group was considered as a negative control. The ulcers in the positive control group were treated by conventional daily dressing using Teriadent (Triamcinolone acetonide 0.1%, Raha Pharmaceutical Co., Isfahan, Iran). For oral ulcer dressing, sterilized curcumin-loaded collagen scaffolds with the specific dimensions (10 mm × 10 mm) were placed into a 48-well culture plate with or without cells, and then incubated at 37 °C and 5% CO_2_ for 24 hr. Ulcers in the third group were covered with the scaffolds that were incubated in a cell-free culture medium while the fourth group was treated with cell-scaffold constructs by seeding density of 3 × 10^6^ cells per scaffold. All curcumin-loaded collagen scaffolds were fixed onto the oral ulcers by 5-0 nylon sutures (Supa, Iran). Treatment modalities were started immediately after the ulcer creation (day 0). To avoid the exacerbation of experimental oral ulcers or removing scaffold from the ulcer site, the rats were fed with sugar-containing water during the experiment. On 3^rd^ and 7^th^ day after ulceration, half of the rats in each group (n=5) were sacrificed in a chamber with rising concentrations of CO_2_. In each rat, the full-thickness excisional biopsies were taken from both sides of the buccal mucosa and subjected to histopathological examination (right side) as well as measurement of the tissue levels of MPO, SOD, and IL-1β production (left side).

***Histopathological assessment***

After neutral-buffered formalin fixation, specimens were processed using the standard processing machine (DID SABZ Co., Iran) and then embedded into paraffin blocks. Serial sections of 5-μm thickness were cut using a microtome, deparaffinized, rehydrated, and stained with hematoxylin-eosin (Merck, Germany). Slides were examined *via* a light microscope by an experienced observer blinded to treatments. Finally, each histological feature was scored according to the following criteria (33):

Score 0: normal epithelium and connective tissue without vasodilatation; absence of or discreet cellular infiltration; absence of hemorrhagic areas, ulcerations, or abscesses.

Score 1: discreet vascular ingurgitation, re-epithelization areas; discreet inflammatory infiltration with mononuclear prevalence; absence of hemorrhagic areas, edema, ulcerations, or abscesses.

Score 2: moderate vascular ingurgitation, areas of hydropic epithelial degeneration, inflammatory infiltration with neutrophil prevalence, presence of hemorrhagic areas, edema and eventual ulcerations, absence of abscesses.

Score 3: severe vascular ingurgitation and dilatation, inflammatory infiltration with neutrophil prevalence, presence of hemorrhagic areas, edema, and extensive ulceration and abscesses.

***The MPO, SOD and IL-1β activities***

Surgical biopsies from the oral mucosa (10–20 mg) were cut into small pieces and homogenized in PBS. The homogenate was centrifuged at 12,000 rpm for 15 min at 4 °C to obtain the supernatant for the subsequent analyses. MPO and SOD enzyme activities were determined by commercially available kits (Kiazist Life Sciences, Tehran, Iran) through the spectrophotometric kinetic assays at 405 nm and 570 nm, respectively. Furthermore, the quantitative measurements of IL-1β in oral mucosa biopsies were performed using an enzyme-linked immunosorbent assay (Abcam, MA, USA).

***Statistical analysis***

All statistical analyses were carried out using the SPSS software package (SPSS Inc., Chicago, USA). Continuous and normally distributed data are expressed as mean ± standard deviation. One-way analysis of variance (ANOVA) and *post-hoc* Tukey’s tests were used to compare the differences among experimental groups. A *P-*value of less than 0.05 was considered statistically significant.

## Results

***Mechanical and Structural Properties of Scaffolds***

**Figure 1:**
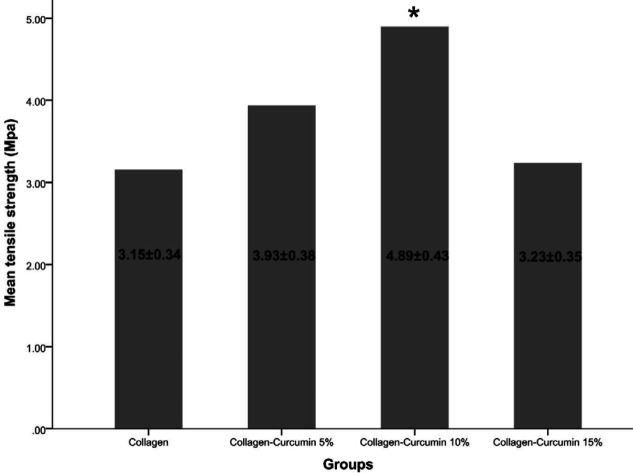
Tensile strength of the investigated scaffolds. The collagen scaffold showed the least tensile strength, and the mean tensile strength of collagen-curcumin 10% was significantly higher than that observed in the curcumin-free collagen scaffold and the collagen-curcumin 15% scaffold. Asterisk (*) indicates a significant difference (*P*<0.05) compared with the collagen group

The mean tensile strengths of collagen, collagen-curcumin 5%, collagen-curcumin 10%, and collagen-curcumin 15% were 3.15±0.34, 3.93±0.38, 4.89±0.43, and 3.23±0.35, respectively. The collagen-curcumin 10% showed significant differences in comparison with the curcumin-free collagen scaffold (*P*=0.0009) and the collagen-curcumin 15% scaffold (*P*=0.011). Experimental findings showed that the collagen scaffold in combination with various concentrations of curcumin could determine the mechanical properties of the structure and improve its tensile strength. However, the curcumin-free collagen scaffold exhibited the least strength as expected (Figure 1). Our results showed that the curcumin concentrations higher than 10% had negative effects on the mechanical properties of the collagen scaffold; therefore, the curcumin concentrations with desirable mechanical properties (5% and 10%) were selected for the downstream processes.

**Figure 2:**
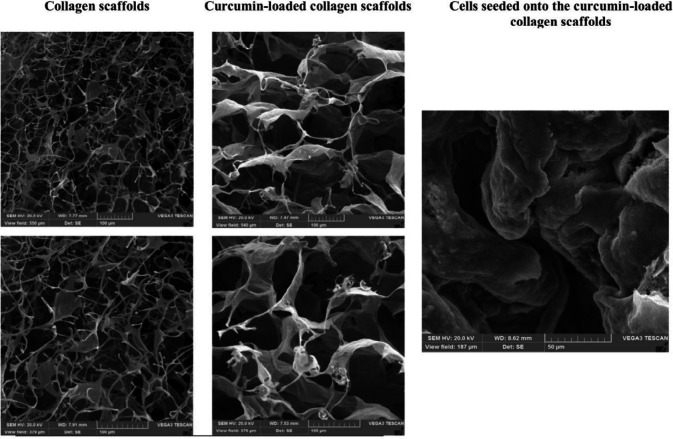
The morphology of collagen scaffolds, curcumin-loaded collagen scaffolds, and ADSCs seeded onto the curcumin-loaded collagen scaffolds after 10 days of cell seeding by SEM. Addition of curcumin to collagen scaffolds could significantly increase the pore size of the structure

ADSCs: adipose tissue-derived stem cells; SEM: scanning electron microscopy

The porosity of different structures was measured *via* their SEM images. Our results revealed that the porosity of all curcumin-free and curcumin-loaded collagen scaffolds was more than 75%. Although the collagen scaffolds showed a highly porous structure, the curcumin-loaded collagen scaffolds demonstrated the higher porosities (76.58±1.8 *vs.* 79.67±1.5; *P*=0.029). Furthermore, the mean pore size of collagen scaffolds was significantly increased after addition of curcumin in comparison with curcumin-free collagen scaffolds (94±26 *vs.* 52±12 µm; *P*=0.034). The obtained porosities for each group, the mean pore sizes, as well as the morphology of the scaffolds in different scales, are presented in Figure 2.

***Characterization of ADSCs***

**Figure 3:**
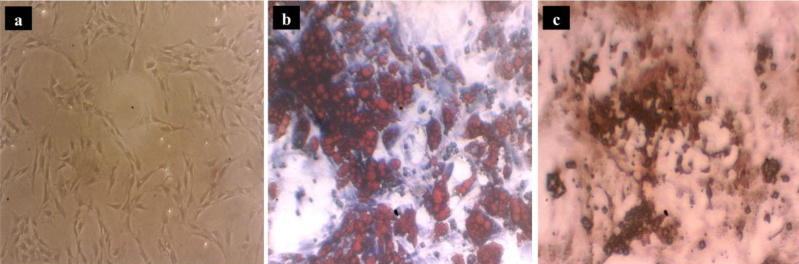
Characterization of ADSCs. The plastic-adherent proliferation and typical cell morphology (a). Differentiation of ADSCs into adipocytes by staining the intracellular lipid droplets with Oil Red-O after 21 days of cultivation (b). Differentiation of ADSCs into osteoblasts by Alizarin Red staining after 21 days of cultivation (c)

**Figure 4:**
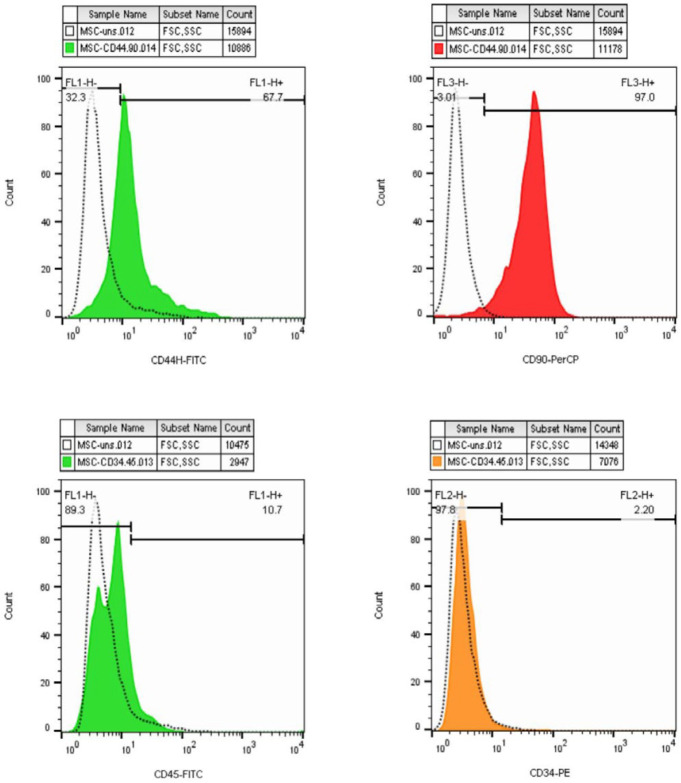
Flow cytometric analysis of ADSCs surface markers expression. ADSCs positively expressed CD44 and CD90 and exhibited negative expression for CD34 and CD45

ADSCs were confirmed by plastic-adherent proliferation and typical cell morphology (Figure 3a). ADSCs possess the potential to differentiate into multiple cell lineages including mature adipocytes and osteoblasts. Adipogenesis was confirmed by staining the intracellular lipid droplets with Oil Red-O after 21 days of cultivation in the induction medium (Figure 3b). Regarding osteogenic differentiation property, the presence of calcium deposits was assessed using Alizarin Red staining after 21 days (Figure 3c). Furthermore, a variety of markers were evaluated by a flowcytometric approach to identify ADSCs. Here, we found ADSCs (at ≥ passage 4) positively expressed specific cell surface markers including CD44 and CD90 as mesenchymal markers and exhibited negative expression for CD34 and CD45 as hematopoietic markers (Figure 4).

***MTT assay***

**Figure 5:**
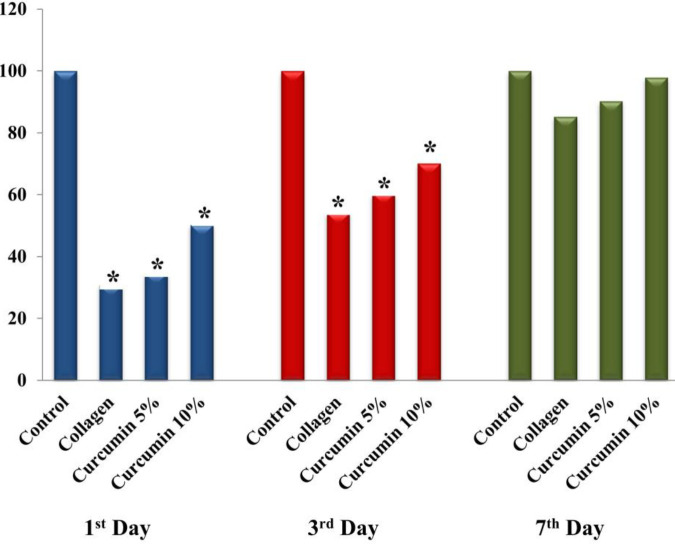
Evaluating the viability of ADSCs by MTT assay. Compared with the control group, the viability of ADSCs in the collagen, curcumin 5%, and curcumin 10% treated groups showed significant reduction at the 1^st^ and 3^rd^-day follow-ups; however, no significant difference was observed in the cell viability between experimental groups at the 7^th^ day. Asterisk (*) shows a significant difference (*P*<0.05) compared with the control group

To investigate the effects of curcumin on ADSCs, the viability of cells after exposure to different curcumin concentrations was evaluated by MTT assay. The relative cell viability in different groups was compared with the control group at 1^st^, 3^rd^, and 7^th^ day post-exposure. Our results represent a significant reduction in the viability of ADSCs at 1^st^ and 3^rd^-day post-exposure in the collagen (*P*<0.0001 and *P*<0.0001), curcumin 5% (*P*=0.001 and *P*<0.0001), and curcumin 10% (*P*=0.003 and *P*<0.0001) treated groups in comparison with the control group (Figure 5). However, no significant difference was observed in cell viability at 7^th^ day post-exposure in the collagen (*P*=0.069), curcumin 5% (*P*=0.273), and curcumin 10% (*P*=0.973) treated groups in comparison with the control group.

***Tissue inflammation, oxidative response, and cytokine level***

The MPO, SOD, and IL-1β activities were measured in the full-thickness excisional biopsies of oral ulcers (Table 1). The results showed that MPO and IL-1β levels were significantly reduced in all experimental groups in comparison with the negative control group (*P*<0.05). However, there was no significant difference in the mean MPO and IL-1β levels between the positive control and curcumin-loaded collagen scaffold groups (*P*>0.05).

The SOD activity was significantly elevated in all experimental groups compared with the negative control group (Table 1). The tissue SOD activity did not show any significant difference between positive control and curcumin-loaded collagen scaffold groups (*P*>0.05). Among all groups, the lowest MPO and IL-1β levels, as well as the highest SOD activity, were found in the group in which ADSCs at the density of 3 × 10^6^ cells were seeded onto a curcumin-loaded collagen scaffold.

***Histopathological findings***

**Figure 6:**
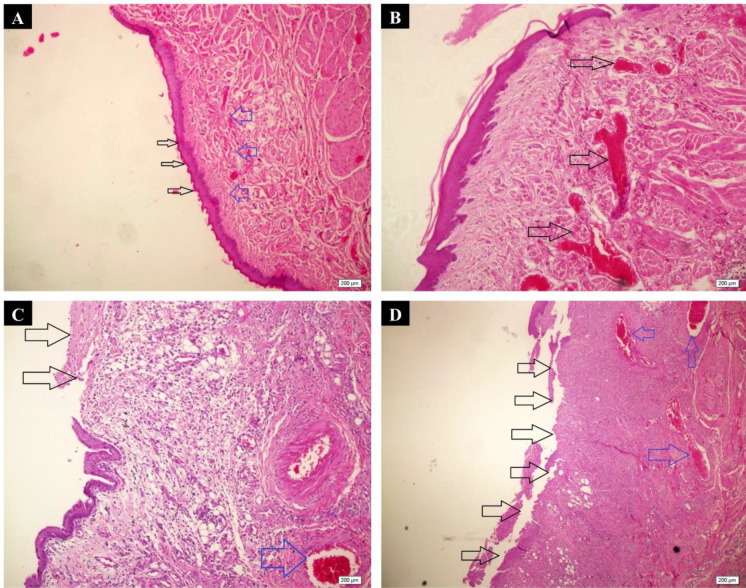
Microscopic analysis of oral ulcer healing using the H & E staining. A) Normal epithelium (black arrows) and connective tissue without vasodilation, absence of discrete cellular infiltration, and hemorrhagic areas corresponding to Score 0 (H & E; ×100). B) Discrete vascular ingurgitation (arrows), re-epithelialization, and inflammation with mononuclear prevalence corresponding to Score 1 (H & E; ×100). C) The eventual ulceration (black arrows), presence of inflammatory infiltration, and moderate vascular ingurgitation (blue arrow) corresponding to Score 2 (H & E; ×100). D) Severe vascular ingurgitation and dilation (blue arrows), inflammatory infiltration with neutrophilic prevalence, and extensive ulceration (black arrows) corresponding to Score 3 (H & E; ×40)

The histopathological evaluation of ulcer healing was performed at 3^rd^ and 7^th^ days post ulceration (Table 2 and Figure 6). From the microscopic point of view, there were no significant differences in the rate of healing between the negative control, positive control, and curcumin-loaded collagen scaffold groups (*P*>0.05), neither at the 3^rd^ day nor at the 7^th^ day follow-up. However, the group in which ADSCs were seeded onto a curcumin-loaded collagen scaffold exhibited a more substantial degree of ulcer healing at 3^rd^ and 7^th^ days follow-up only when compared with the negative control group (*P*=0.037 and *P*=0.004, respectively).

ADSCs: adipose tissue-derived stem cells

ADSCs: adipose tissue-derived stem cells

ADSCs: adipose tissue-derived stem cells

**Table 1:** MPO, SOD and IL-1β activities in experimental groups

**Sampling time**	**Parameters**	**Experimental groups**			
		**Negative control**	**Positive control**	**Curcumin-loaded collagen scaffold**	**ADSCs seeded onto a collagen-curcumin scaffold**
**3** ^rd^ ** day**	**MPO (mU/mg)** **mean ± SD**	12.88 ± 0.43	9.88 ± 0.37 ^(*)^	9.80 ± 0.57 ^(*)^	6.72 ± 0.67 ^(*^^†^^Ψ)^
	**SOD (U/mg)** **mean ± SD**	63.02 ± 3.71	89.36 ± 1.41 ^(*)^	89.56 ± 2.27 ^(*)^	101.32 ± 3.46 ^(* † Ψ)^
	**IL-1β (pg/mL)** **mean ± SD**	1134.98 ± 131.87	878.46 ± 37.99 ^(*)^	886.90 ± 26.16 ^(*)^	679.52 ± 33.98 ^(* † Ψ)^
**7** ^th^ ** day**	**MPO (mU/mg)** **mean ± SD**	10.98 ± 0.37	8.06 ± 0.56 ^(*)^	8.10 ± 0.44 ^(*)^	5.92 ± 0.31 ^(* † Ψ)^
	**SOD (U/mg)** **mean ± SD**	74.50 ± 3.41	98.62 ± 5.26 ^(*)^	105.04 ± 5.39 ^(*)^	125.66 ± 5.86 ^(* † Ψ)^
	**IL-1β (pg/mL)** **mean ± SD**	945.98 ± 37.47	691.34 ± 28.65 ^(*)^	708.56 ± 46.70 ^(*)^	558.00 ± 37.41 ^(* † Ψ)^

Asterisk (*), Dagger (†), and Psi (Ψ) represent significant differences (*P*<0.05) with negative control, positive control, and curcumin-loaded collagen scaffold groups, respectively

MPO: myeloperoxidase; SOD: superoxide dismutase

**Table 2:** Histopathological scoring of experimentally induced oral mucosal ulcers in rats

**Sampling time**	**Score**	**Experimental groups**			
		**Negative control**	**Positive control**	**Curcumin-loaded collagen scaffold**	**ADSCs seeded onto a collagen-curcumin scaffold**
**3** ^rd^ ** day**	**Score 0, n (%)**	NF	NF	NF	NF
	**Score 1, n (%)**	NF	NF	NF	1 (20%)
	**Score 2, n (%)**	NF	2 (40%)	2 (40%)	3 (60%)
	**Score 3, n (%)**	5 (100%)	3 (60%)	3 (60%)	1 (20%)
**7** ^th^ ** day**	**Score 0, n (%)**	NF	1 (20%)	1 (20%)	3 (60%)
	**Score 1, n (%)**	NF	2 (40%)	2 (40%)	2 (40%)
	**Score 2, n (%)**	4 (80%)	2 (40%)	2 (40%)	NF
	**Score 3, n (%)**	1 (20%)	NF	NF	NF

NF: not found

## Discussion

Oral ulcers generally occur anywhere in the mouth but more common in non-keratinized mucosal surfaces, such as the lips, buccal mucosa, ventrum of the tongue, and floor of the mouth (2, 3). Ulcer healing in the oral cavity shares similarities with cutaneous wounds; however, they differ in many important aspects. Oral mucosal wounds occur in a warm fluid, resulting in more rapid healing, faster re-epithelialization, less inflammation, and relatively minimal to no scar formation compared with dermal wounds. A successful ulcer healing depends on the collaboration of many cell strains and their products as well as a series of sequential responses including hemostasis, inflammation, proliferation, and remodeling that allow the closure of ruptures in the oral tissue (34, 35).

Conventional management of oral mucosal ulcers has relied on palliative care in combination with antimicrobial therapies that reduce the severity of lesions and the risk of secondary infections (36). However, finding suitable multidisciplinary approaches with fewer side effects to relieve the pain, promote healing, and prevent ulcer recurrence after healing are the main goals of novel treatments of oral ulcers. The field of tissue engineering has provided new solutions for reconstruction of oral mucosa with or without cells. The potential of each of ADSCs (11, 37), curcumin (20, 22, 23), and collagen scaffold (38-40) in the healing of various ulcers have been confirmed in animal models; however, structures fabricated with a combination of these elements would be more effective in promoting wound healing. Therefore, the present study attempted to evaluate the effect of ADSCs seeded onto the curcumin-loaded collagen scaffold on experimental oral ulcers using the histological examination as well as tissue MPO, SOD, and IL-1β assessments.

Overall, the scaffold materials and its fabrication methods represent important components for tissue engineering thus the success of the technique largely depends on it. In the present study, we successfully fabricated a highly porous curcumin-loaded collagen scaffold with a small fiber-like structure that had a promising amount of porosity (more than 75%) for applications in tissue engineering and stem cell-based therapies. Our results revealed that the addition of curcumin to the collagen scaffold can cause tendency of the structure to stick together and form larger units of various shapes and strength; hence, it might be a reason why the curcumin-free collagen scaffold has poor mechanical properties. In line with our results, previous studies found that collagen is a key component of ulcer healing and plays a critical role in hemostasis, inflammation, proliferation, and remodeling. Furthermore, the collagen scaffolds are able to control the release of bioactive molecules as well as the secretion of re-epithelialization and angiogenesis mediators; therefore, the porous collagen scaffold could be used for ulcer healing without further inflammation and adverse effects (16, 19, 38, 41). Although a wide range of curcumin concentrations (5–25%) can be used as an additive material to make scaffolds (24), our results showed that the curcumin concentrations higher than 10% had negative effects on the mechanical properties of the curcumin-loaded collagen scaffold. In line with our findings, Polat and Kinali (42) showed that curcumin concentration of 10 % was no longer able to penetrate the fiber; therefore, the solution viscosity can be considered above the optimum value for fiber formation. A similar trend was observed for the diameter of curcumin-loaded polycaprolactone/chitosan (43), polyurethane (44), and cellulose acetate (45) fibers. In another work, Amirthalingam *et al*. (46) fabricated the novel biocompatible PLGA–curcumin microparticle-embedded chitosan scaffold for wound healing application. Their results indicated that the existent scaffold could be used as a drug delivery system to treat severe and chronic wounds. Another study (20) also showed that the curcumin-loaded chitosan nanoparticles impregnated into collagen-alginate scaffolds can be considered a promising strategy for diabetic wound healing through the combination of synergistic effects. In contrast to our findings, reduced diameter of the fibers with increased curcumin content has also been reported by some researchers (47, 48).

The seed cells are another key component of tissue engineering. In this study, the impacts of ADSCs were investigated to find their potential for seeding on the curcumin-loaded collagen scaffold. Our results demonstrated that ADSCs had the potential to differentiate into multiple lineages upon appropriate stimulation that was confirmed in previous studies (10, 12, 28, 29). Furthermore, ADSCs are able to adhere, proliferate, and migrate to curcumin-loaded collagen scaffolds without affecting their properties. In addition, they are found in abundant quantities and easily harvested from different parts of the body with minimal discomfort, thus ADSCs could be considered a reliable alternative to other sources of stem cells, like bone marrow (12, 49).

In the current study, the lowest MPO and IL-1β levels as well as the highest SOD activity were interestingly found in the cell-seeded scaffold group in comparison with negative control, positive control, and curcumin-loaded collagen scaffold groups. The positive control and curcumin-loaded collagen scaffold groups had the same levels of MPO, SOD, and IL-1β; however, they showed significant differences in comparison with the negative control group. Generally, the body produces free radicals during its metabolic processes, immune reactions, and tissue repair; however, the normal cells possess enzymatic and non-enzymatic anti-oxidants to protect them from oxidative stress. An increased generation of intracellular free radicals over the physiological value might lead to cell damage and plays an important role in delayed and impaired ulcer healing as well as poor outcomes (50, 51).

In order to avoid any oxidative injury, endogenous anti-oxidants, such as SOD have been discovered to control the intracellular level of ROS through their signaling pathways (52). MPO is another enzyme that is generally found in neutrophils and considered a quantitative index of inflammatory infiltration in both acute and chronic conditions. Activation of neutrophils and facilitating their chemotaxis towards the wound site can worsen ROS production, resulting in tissue damage and delayed wound healing (53, 54). The inflammatory phase is an essential component of the healing process that occurs within the first hours after wounding. Prolonged inflammatory responses can increase the release of pro-inflammatory cytokines such as IL-1β, which is expressed by many cells. In particular, the high level of IL-1β correlates with non-healing ulcers, severe wound healing disturbances, and scar formation. The pro-inflammatory events can initiate through binding of IL-1β to its receptor (IL-1R); therefore, the competitive inhibition of this signaling function by an immunosuppressive cytokine, called IL-1Ra, might protect the tissues from inflammation-induced injuries (55-57). In line with our findings, several studies have suggested that curcumin is a potent anti-inflammatory and anti-oxidant component (58) with the ability to increase SOD activity (59) and attenuate the expression of IL-1β (60, 61). Study (37) showed that curcumin can inhibit the release of MPO by activated neutrophils which consequently leads to lower neutrophil accumulation and ROS production as well as accelerated healing process. Furthermore, previous studies showed that ADSCs were crucially important for the attenuation of inflammation and accelerating wound healing *via* regulation of the inflammatory response and secretion of anti-inflammatory cytokines. However, the exact mechanism by which ADSCs mediate inflammation remains unclear (62-64). Researchers (23) showed smaller oral ulcer size and faster healing in rats following the administration of curcumin, which might be due to increased levels of TGF-β and α-SMA. 

Our data also correlated with the histological findings of oral ulcer healing. Our histopathological examinations showed an accelerated healing process at 3^rd^ and 7^th^ days post ulceration in the group treated with ADSCs seeded onto the curcumin-loaded collagen scaffold in comparison with the untreated control group. It seems the synergistic effects of curcumin, collagen scaffold, and ADSCs were responsible for the better healing of oral ulcers in this group. In line with our findings, several studies also showed the healing effects of curcumin, collagen, and stem cells in animal models. Topical application of curcumin in an animal oral ulcer model (New Zealand white rabbits) exhibited accelerated wound healing on day 7 in the curcumin-treated group in comparison with the control group, which was characterized by smaller ulcer size, less inflammatory cell infiltration, and incomplete re-epithelialization (22). Amirthalingam *et al*. (46) showed that the excision wound in rats healed significantly on the 8^th^ day in the PLGA–curcumin microparticles treated groups but not the control group. The PLGA–curcumin microparticles provided faster action by contracting the wound in the initial stage of wound healing. Another study (65) evaluated the healing effects of locally injected platelet-rich plasma (PRP) and bone marrow-derived mesenchymal stem cells (BMSCs) on oral ulcer and found that both PRP and BMSCs accelerated wound healing and enhanced the quality of the healing tissue with the latter being slightly more effective and faster. The wound healing response in rabbit oral lesions grafted with acellular collagen scaffolds also showed a significantly lower contraction of wounds in comparison with their controls, suggesting that collagen scaffolds-grafted wounds favor regeneration of oral mucosa (41).

## Conclusion

In this study, we have successfully applied ADSCs seeded onto the curcumin-loaded collagen scaffold for reconstruction of oral mucosa in an experimental rat model. Our findings showed this structure can promote the healing process of oral ulcers in rats and markedly reduced inflammatory responses. Due to similarities with the oral mucosa of humans, rats have been extensively used to investigate the wound healing process in oral structures and tissues. The morphology of the curcumin-loaded collagen scaffold indicated a highly porous structure and its porosity was very high.
